# Should I stay or should I go? Individual movement decisions during group departures in red-fronted lemurs

**DOI:** 10.1098/rsos.180991

**Published:** 2019-03-20

**Authors:** Anna Lucia Sperber, Peter M. Kappeler, Claudia Fichtel

**Affiliations:** 1 Behavioral Ecology and Sociobiology Unit, German Primate Center Leibniz Institute for Primate Research, Kellnerweg 4, 37077 Göttingen, Germany; 2 Department of Sociobiology/Anthropology, Johann-Friedrich-Blumenbach Institute for Zoology and Anthropology, University Göttingen, Kellnerweg 6, 37077 Göttingen, Germany; 3 Leibniz ScienceCampus ‘Primate Cognition’, Kellnerweg 4, 37077 Göttingen, Germany

**Keywords:** group coordination, collective movements, mimetism, primates, *Eulemur*

## Abstract

Collective movements are essential for maintaining group cohesion. However, group members can have different optimal departure times, depending on individual, social and contextual factors whose relative importance remains poorly known. We, therefore, studied collective departures in four groups of red-fronted lemurs (*Eulemur rufifrons*) in Kirindy Forest, Madagascar, to investigate the influence of an individual's age, sex, their affiliative relationships and their proximity to other group members at the time of departure on their individual departure decision. We recorded behavioural and spatial data on individual departures during 167 group movements and conducted group scans (181–279 per group) to assess affiliative relationships. All factors influenced individual departures. Both affiliation and proximity determined a mimetic joining process in which dyads with stronger affiliative bonds departed in closer succession, and individuals followed the initiator and predecessors more quickly when they were in closer proximity at departure. While the influence of affiliation is common, the effect of inter-individual distance has rarely been considered in groups with heterogeneous social relationships. Although local rules influenced joining, the overall movement pattern was mainly determined by individual traits: juveniles took protected central positions, while females made up the van and males brought up the rear. Individual needs, expressed in the departure order, to an extent overruled the effect of affiliation. These results highlight the importance of considering individual, social and contextual factors collectively in the study of collective movements.

## 1. Introduction

Living in a group provides benefits [1–5], such as a reduced individual predation risk [6–8], reduced time spent on vigilance [9] and more efficient foraging, e.g. through information transmission [10–12]. To maintain group cohesion, animals normally do not decide independently on activity changes, but rather base their choice on the actions of their group mates. This form of positive feedback, when one individual taking an action makes it more likely for others to do so as well, is called mimetism [13]. Mimetism has been suggested in several species to determine the joining process of collective departures [14,15]. Mimetism can be anonymous, where joining solely depends on the number of individuals who have already left [16], or it can be selective. Joining can be shaped by affiliation in that the strength of the social bond between individuals determines how much one's actions are influenced by other's. Affiliative mimetism has been shown to determine following in primates [17–22], ungulates [23–26] and birds [27]. Joining can also be based on distance, so that individuals are more likely to join a group movement when those in closest proximity to themselves do so. This mechanism has been demonstrated in fish [28], domestic geese (*Anser domesticus*) [29], domestic sheep (*Ovis aries*) [23] and anonymous human groups [30]. It has been termed ‘local mimetism’ by Ward *et al.* [28]. However, since anonymous and affiliative mimetism are also considered local rules [17,20], we feel that the word ‘local’ is ambiguous here. We prefer the term ‘spatial mimetism’ to refer to mimetism based on physical proximity, to emphasize the spatial effect and to clarify that all kinds of mimetism, not only spatial mimetism, can be considered a local rule.

In groups where individuals exhibit heterogeneous social relationships, the effects of affiliation and proximity on joining are hard to discern, as proximity is generally necessary for most affiliative behaviours (for an exception see [31]). In chacma baboons (*Papio ursinus*), for example, affiliation and spatial association determined the likelihood of individuals following each other [20]. The spatial association considered in this study on baboons was, however, the proportion of time individuals spent in proximity as nearest neighbours throughout the observation period, making it difficult to distinguish the effects of social affiliation and spatial association. Although most of these studies examined the occurrence of mimetism on a dyadic level, this does not mean that individuals base their decision solely on individuals closest to them or with whom they have stronger social bonds. As highlighted in olive baboons (*Papio anubis*) through high-resolution GPS tracking data, individuals indeed base their movement decisions on several neighbouring individuals [32].

In general, an individual's departure time relative to the other group members' determines their position in the movement order. Since different positions have different advantages and disadvantages, the decision on when to leave is also influenced by individual, non-mutually independent factors, such as vulnerability and nutritional needs [33], which largely depend on the individual's age and sex. Moving groups often display a ‘protective travel order’ [34], with the most vulnerable group members in the centre. Since the van and rear of a group are exposed to a higher average predation risk, less vulnerable individuals may serve as a van- and rearguard (e.g. [35]). The van is a high-risk but also a high-payoff position. Due to the ‘finder's advantage’ [39], specific or high nutritional needs [37–39] can be met more easily in this position [40–44].

Since individual, social and contextual factors can all affect the choice of departure time, they have to be considered together to understand departure decisions in collective movements. However, only a few studies of collective departures have considered contextual factors, such as the spatial positions of group members, at all (for an overview of those studies that have included a spatial factor, table 3). It is even rarer that studies investigated the effects of both proximity and affiliative relationships on following, and when they did, the measures were often conflated. Furthermore, these local rules are usually considered separately from the global effect of ecological factors, as expressed in an individual's position in the departure order.

Thus, to investigate the interacting effects of individual, social and contextual factors on individual departure decisions, we studied group departures in wild red-fronted lemurs (*Eulemur rufifrons*). Red-fronted lemurs live in small multi-male multi-female groups and are relatively egalitarian and socially tolerant Malagasy primates [45]. They are cathemeral and arboreal but travel and forage regularly on the ground. Adult females lead group movements most frequently, but leadership is variable [46,47]. As group living in lemurs is the result of convergent evolution with other primate taxa [48], studying lemur group coordination processes provides a valuable comparative perspective to begin identifying general principles in this context, at least for primates. We, therefore, examined the influence of age, sex, proximity at departure and affiliative relationships on individual departure decisions in collective movements. If red-fronted lemurs exhibit affiliative mimetism in group departures, we predicted individuals would preferentially follow those group members they were more strongly affiliated with. By contrast, we predicted individuals would preferentially follow those in closer spatial proximity if following is guided by spatial mimetism. If differences in needs affect individual departure decisions, we predicted red-fronted lemurs of different ages and sex to take up different positions in the departure order corresponding to the positions' advantages and disadvantages.

## 2. Material and methods

### 2.1. Study site and subjects

The study was conducted at the field site of the German Primate Center in Kirindy Forest, Western Madagascar. The 60 ha study site is situated in a dry, semi-deciduous forest within a forestry concession operated by the Centre National de Formation, d'Etudes et de Recherche en Environnement et Foresterie [49]. Several natural predators of red-fronted lemurs are present at this site. We studied four groups of red-fronted lemurs, with a total of 31 individuals (group A: 3 females, 3 males, 2 juveniles; group B: 2 f, 2 m, 2 j; group F: 2 f, 2 m, 2 j; group J: 4 f, 3 m, 4 j). Study animals are individually marked and are well habituated to observers, as they are part of a long-term study [50].

### 2.2. Data collection

Data were collected from April to September 2014. Observations were conducted mainly between 8.00–11.00 and 14.00–17.00 h, since groups usually rest during midday [51]. We observed one focal group per half-day, and morning and afternoon observation sessions were balanced among groups. Observations were recorded with a handheld computer (Psion Workabout Pro 3).

#### 2.2.1. Group departures

Group departures were recorded with all-occurrences sampling, following the definitions of Pyritz *et al*. [47]. An initiator was defined as an individual who after being ‘stationary for ≥ 4 min moved ≥ 15 m away from [the other] group members in a directed manner without pausing’ [47, p. 1332]. An initiation attempt was considered successful if at least one follower (apart from dependent offspring) was recruited. A follower was defined as an individual who left less than or equal to 10 min after the initiator and whose ‘movement diverged ≤ 45° from the trajectory of the movement of the initiator’ [47, p. 1332].

We disregarded group movements caused by mating or intergroup conflicts. We treated all group movements as independent events for statistical analyses (*n* = 167 successful group movements, group A: *N* = 49, group B: *N* = 37, group F: *N* = 35, group J: *N* = 46). Failed initiations, where no followers were recruited and the initiator returned to the group within 10 min, were extremely rare (*n* = 2) and excluded from the analysis. During group departures, we noted the identities of initiators and followers and the timing of individual departures. Whenever possible, we also recorded the distances between the initiator and the other group members at the time of initiation and the distances between followers succeeding each other. The distance was estimated in five categories: 0–1 m, 1–3 m, 3–5 m, 5–10 m and greater than 10 m.

To quantify the travel association of each dyad, we calculated their inter-departure interval (IDI) as their average difference in departure time across all movements, adjusted for the mean difference in departure time per movement (equation (2.1)). A dyad often following each other in close succession would, therefore, have a short IDI.

2.1 $$\mathrm{ID}I_{xy}=\frac{\sum_{i=1}^{n}|\mathrm{departure}\;\mathrm{tim}e_{xi}\;-\;\mathrm{departure}\;\mathrm{tim}e_{yi}|/\mathrm{mean}\;\mathrm{departure}\;\mathrm{time}\;\mathrm{differenc}e_{i}}{n}$$

#### 2.2.2. Affiliation

To characterize affiliative relationships, we conducted group scans every 15 min when the group was stationary and recorded the activity and social interactions of all visible animals. Grooming and huddling were used as measures of affiliation. Grooming was defined as at least one individual grooming another (repeated strokes over the partner's pelage with the toothcomb and/or tongue) or both individuals grooming each other simultaneously. Huddling was defined as the individuals being in contact with their torsos or head to torso in a resting context. For each dyad, we calculated the proportion of scans in which they were grooming or huddling, corrected for the number of scans in which both individuals were observed (number of scans: group A: total 199 scans (ranging from 130 to 172 per dyad), group B: total 200 scans (ranging from 160 to 187 per dyad), group F: total 181 scans (ranging from 134 to 167 per dyad), group J: total 279 scans (ranging from 166 to 206 per dyad). As an index of a dyad's affiliation, we calculated the dyadic composite sociability index (DSI, equation (2.2)) following Silk *et al*. [52].

2.2 $$\mathrm{DS}I_{xy}=\frac{\left((\mathrm{groomin}g_{xy}/\mathrm{groomin}g_{\mathrm{group}\;\mathrm{mean}})+(\mathrm{huddlin}g_{xy}/\mathrm{huddlin}g_{\mathrm{group}\;\mathrm{mean}})\right)}{2}.$$

### 2.3. Statistical analyses

All analyses were conducted in R v. 3.0.2 [53]. Frequentist linear models were fit using the package *lmerTest* [54,55] and were chosen according to lowest Akaike information criterion [56]. Bayesian linear models were fit using the package *MCMCglmm* [57] and chosen according to lowest deviance information criterion [58]. The significance level was set at 0.05, and results from the linear models were corrected for multiple testing using the Holm correction [59], unless they were compared using a multiple comparison procedure.

#### 2.3.1. Group cohesion

To characterize the degree of group cohesion, we calculated the median latency to join the movement. Furthermore, we analysed the distribution of the number of departures according to the number of followers (how often how many individuals followed the initiator) within groups, using a χ^2^-test. A heterogeneous distribution of the frequency of number of followers would indicate an all-or-nothing process and a cohesive group, whereas a homogeneous distribution would be caused by clustering into subgroups [60]. We also estimated the effect of group size on the time until the last follower joined the movement, considering only departures in which the entire group followed (*n* = 106). We fit an LMM with the last follower latency as the response, group size as a fixed factor and initiator and last follower ID as random factors.

#### 2.3.2. Individual factors

To assess the influence of individual factors on departure order, we tested the distribution of age-sex classes (juveniles (less than 2.5 years), adult males and adult females) across the departure order. To be able to combine the data from differently sized groups, we grouped departure ranks into the position categories ‘van’, ‘centre’ and ‘rear’. Van and rear were defined as the two first and two last movers, respectively, whereas the size of the centre category differed between groups (range 2–7 individuals; electronic supplementary material, figures S1 and S2). We furthermore included the option ‘not joined’ as a position category for the departures in which an individual did not participate in the movement. We fit a linear model for the number of times an individual had been observed in each position category divided by the individual's total number of departures. We excluded the intercept to allow for an easier comparison of the effects [61,62] and included group and a factor combining age-sex class and position category as fixed factors. We then used multiple comparisons (package *multcomp* [63]) to test for differences between juveniles, adult males and adult females within position categories.

#### 2.3.3. Interactions between individual, social and contextual factors

If individuals of the same age class or sex travel together, this assortativity could be due to similar requirements regarding nutrition or safety from predation. However, it could also be caused by affiliative or spatial mimetism, if affiliative bonds and proximity to the initiator are not random with regard to the age class and sex of an individual. To discern these effects, we tested for interactions between these factors.

To determine the effects of individual traits on affiliative bonds, we calculated the weighted assortativity coefficient for age-sex classes (juveniles, adult males and adult females) in the affiliation network of each group using the package *assortnet* [64]. To examine the effect of an animal's age-sex class on its proximity to the initiator at initiation, we fit an LM with an individual's number of observations in a distance category divided by their overall number of observations as the response (*n* = 237 observations). We included group and a factor combining age-sex class and distance category as fixed factors and excluded the intercept to allow for an easier comparison of the effects. We used multiple comparisons (package *multcomp* [63]) to test for differences between age-sex classes in their occurrence in the distance categories 0–1 m and greater than 10 m, since differences in occurrence in these extremes of the recorded range would have the biggest effect.

#### 2.3.4. Variation in affiliation strength

We assessed how evenly distributed affiliative contacts were across group members by inspecting the distribution of the DSI across dyads (*n* = 113). The variation in relationship strength between dyads is expressed in the skewness of the distribution curve, which can be gauged by comparing the curve's mean and median [65–67].

#### 2.3.5. Social and contextual factors

Our aim was to assess the influence of affiliation and proximity on following behaviour. To control for a potential covariation between the two factors and to determine other influences on proximity, we fit a Bayesian GLMM, regressing a dyad's proximity at departure on their DSI and the age-sex class of the individuals (juvenile–juvenile, female–female, female–male, female–juvenile, male–male, male–juvenile). We included group as a fixed factor, event ID as a random factor and dyad ID as a multiple membership random factor. The multiple membership approach was chosen to acknowledge the fact that each individual was part of several dyads. The model contained both the distance to the initiator and to the predecessor (*n* = 347 observations).

Since affiliation and proximity at departure were not independent, we assessed the effects of affiliation and proximity separately. This allowed us to make use of the much bigger sample size for affiliation and to choose a measure which probably better represents the way a factor would affect following. One problem in testing for an influence on followership is that the animal an individual is closest to in departure time may not actually be the one it is following. For affiliation, we addressed this problem by inspecting the IDIs for all group members. This was possible since data on affiliation were available for all dyads and were naturally the same for all departures. A similar approach could not be used for proximity, however, since recording the distances between all group members was not always possible due to the quick succession of followers. We, therefore, restricted our data collection and analyses to the distance of followers to the initiator and to the direct predecessor.

The effect of affiliation on following was tested as the influence of the DSI of a dyad on their IDI. We fit a Bayesian GLMM on data from all four groups (*n* = 113 dyads), regressing dyad IDI on dyad DSI. We included group as a fixed factor and dyad as a multiple membership random factor. The age-sex classes of a dyad were also included as a random factor.

To assess the influence of spatial proximity on following, we fit two LMMs regressing the latency in following an individual on the proximity to it. The response was the following latency, divided by the mean of the following latencies of the departure event (square-root transformed in the first model and log-transformed in the second model). In the first model, the latency considered was the latency in following the predecessor (including the initiator). We included the distance to the predecessor and the age-sex class (juvenile, adult female, adult male) of the follower as fixed factors and predecessor ID, follower ID, event ID as well as number of predecessors nested in group size as random factors. In the second model, the latency considered was the latency in following the initiator. We included the distance to the initiator and the age-sex class of the follower as fixed factors and initiator ID, follower ID, event ID as well as group size as random factors.

To avoid a biased sample, we only included departures in the distance analysis for which the distance data for all followers had been recorded. Due to the difficult observation conditions with agile animals in a forest setting, applying this criterion greatly reduced our dataset. For the distance to the predecessor, we retained 234 following events in 46 departures and for distance to the initiator we retained 159 following events in 33 departures.

## 3. Results

### 3.1. Strong group cohesion

**Figure 1.**
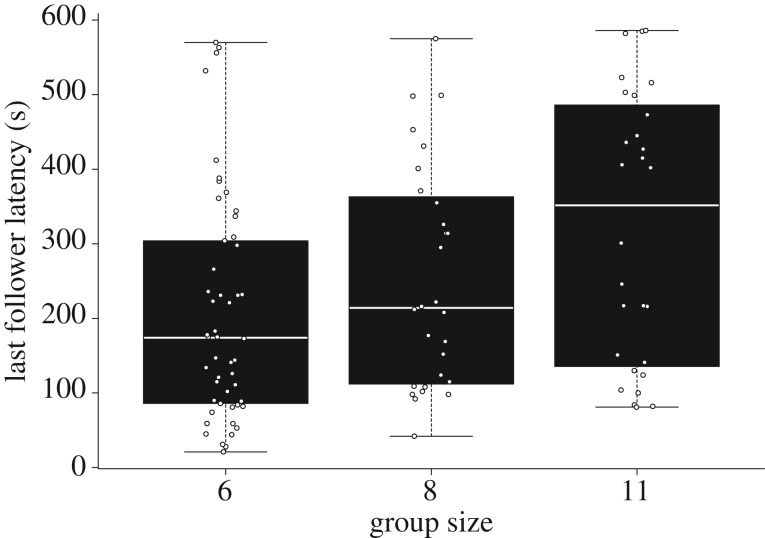
Boxplots with datapoints of joining latencies of the last followers as a function of group size. Group size 6 includes groups B and F. Only movements in which the whole group followed were considered (*n* = 106 movements; group A = 28, B = 25, F = 25, J = 28).

**Figure 2.**
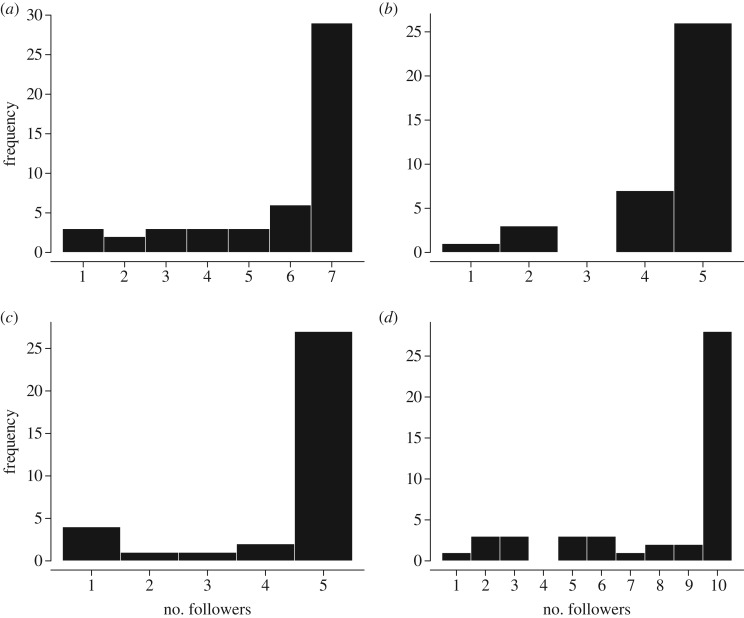
Frequency of follower numbers in group departures in groups A (*a*), B (*b*), F (*c*) and J (*d*). Most successful initiations recruited the entire group.

The median latency in joining the initiator was 96 s (IQR 33–217 s). The latency from initiation to the last follower increased with group size (estimate + s.e.: 22.86 + 7.50, d.f. = 18.66, *t* = 3.05, *p* = 0.007, test against null model: *χ*^2^ = 7.57, d.f. = 1, *p* = 0.006, figure 1). The number of followers recruited per movement was not homogeneously distributed (group A: *χ*^2^ = 82, d.f. = 6; group B: *χ*^2^ = 42.46, d.f. = 3; group F: *χ*^2^ = 72.29, d.f. = 4; group J: *χ*^2^ = 116.39, d.f. = 8; *p* < 0.0001 for all groups), but left-skewed (figure 2). Hence, most of the successful initiations of group movements (64.67%) recruited the entire group. Failed initiations, where no followers were recruited and the initiator returned to the group within 10 min, were extremely rare (on average less than 1% of all observed initiations).

### 3.2. Age class and sex affect departure position

**Figure 3.**
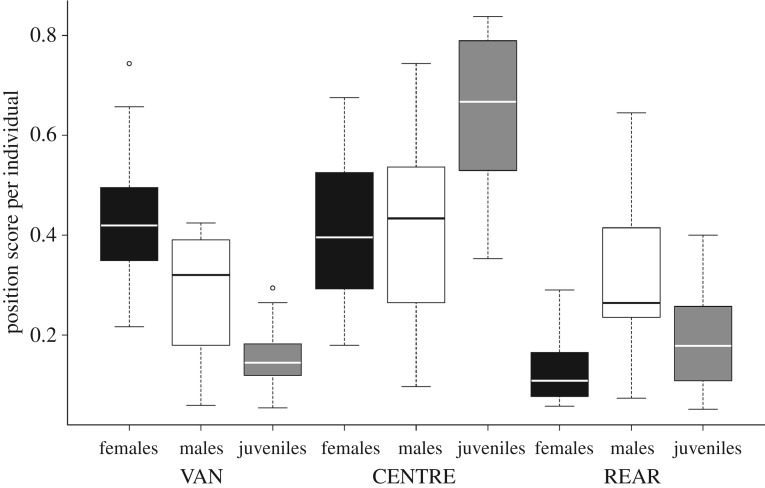
Boxplots of departure positions based on age and sex. Position scores were calculated as the observations of an individual in a position divided by the total number of times the individual was observed in a movement. Females are depicted in black, males in white and juveniles in grey.

Juveniles, adult males and adult females differed significantly in their departure positions. Females were observed significantly more often in the van of the departing group than juveniles and males. The females' distribution on departure ranks (electronic supplementary material, figure S2) shows that females are not only more often in the van because they initiate group movements most frequently, but that they generally cluster on the lower ranks and thus make up the whole vanguard of a movement. In the centre, juveniles were observed more often than both adult males and females. Males were observed significantly more often in the rear than females, but not more often than juveniles (figure 3; electronic supplementary material, table S2).

The assortativity by age class and sex in the departure order cannot result solely from affiliation or proximity. The affiliation networks were not assorted by age-sex classes; instead, they tended towards disassortativity (electronic supplementary material, table S3). Regarding proximity, age classes and sexes did not differ in their occurrence in the 0–1 m distance category from the initiator, nor age classes in the greater than 10 m distance category. However, adult males were observed proportionally more often than adult females in the greater than 10 m distance category from the initiator (electronic supplementary material, table S4).

### 3.3. Affiliative and spatial mimetism influence joining

The distribution of the DSI across dyads was strongly right-skewed (electronic supplementary material, figure S4), as demonstrated by the large difference between the mean of 1 (by definition of the DSI) and the median of 0.38. Furthermore, only 32% (36) of the dyads had DSI values larger than the mean. These results indicate that dyads differed strongly in the strength of affiliative bonds. Dyads with a higher DSI were observed at closer proximity at departure (post. mean: −0.61, CI: −1.10 to −0.20, ESS: 45.5, pMCMC: 0.002), whereas the age-sex class combination of the dyad had no effect on the proximity (electronic supplementary material, figure S3 and table S1).

**Figure 4.**
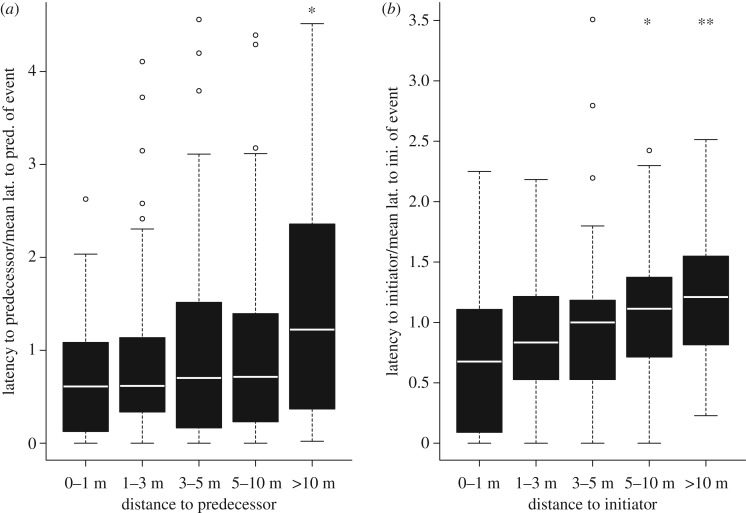
Latency in following the preceding animal (*a*) or the initiator (*b*) as a function of distance. The latency was calculated as the latency of an individual following its predecessor (*a*) or the initiator (*b*) divided by the mean latency of group members following their predecessor or the initiator of the movement event. Distance categories marked with asterisks differ significantly from the 0–1 m category (*p < 0.05, **p < 0.01).

The affiliation of a dyad had a significant effect on their IDI. A higher DSI was associated with a shorter IDI (post. mean: −0.02, CI: −0.04 to −0.01, ESS: 1000, pMCMC: 0.002, table 1). Distance increased the following latency (table 2). The latency to follow the predecessor was significantly higher at greater than 10 m distance than in 0–1 m distance to the predecessor (figure 4*a*). The latency to follow the initiator was significantly higher at 5–10 m and greater than 10 m distance than in 0–1 m distance to the initiator (figure 4*b*). Juveniles followed their predecessors significantly more quickly than adult females, whereas age and sex of the follower had no influence on the latency to follow the initiator.

**Table 1.** Results for the MCMC GLMM regressing a dyad's inter-departure interval on its dyadic composite sociability index (DSI).

	post. mean	CI lower	CI upper	eff. sample size	*p* MCMC
(intercept)	1.01	0.80	1.19	1292.00	<0.001
DSI	−0.02	−0.04	−0.01	1000.00	0.002
group B	0.01	−0.25	0.29	1000.00	1.00
group F	0.01	−0.28	0.26	1000.00	1.00
group J	0.01	−0.25	0.21	1000.00	1.00
The reference level is group A.

**Table 2.** Results for the fixed effects from the predecessor and the initiator LMs (distance categories 0–1 m, 1–3 m, 3–5 m, 5–10 m and greater than 10 m).

	estimate	s.e.	d.f.	*t*-value	Pr(>|*t*|)
latency to predecessor					
(intercept)	0.64	0.09	71.28	7.46	<0.001
distance predec 1–3 m	0.11	0.09	210.43	1.14	0.38
distance predec 3–5 m	0.13	0.10	215.48	1.32	0.38
distance predec 5–10 m	0.16	0.10	213.81	1.54	0.38
distance predec >10 m	0.31	0.12	222.07	2.66	0.03
adult female	0.20	0.08	21.81	2.53	0.04
adult male	0.14	0.08	20.35	1.82	0.08
test against null model: *χ*^2^ = 16.53, d.f. = 6, *p* = 0.01 The reference levels are distance predecessor 0–1 m and age-sex category juveniles.					
latency to initiator					
(intercept)	0.50	0.07	70.85	6.79	<0.001
distance ini 1–3 m	0.09	0.08	149.74	1.08	0.28
distance ini 3–5 m	0.18	0.08	146.29	2.13	0.07
distance ini 5–10 m	0.20	0.08	146.91	2.54	0.04
distance ini >10 m	0.25	0.08	150.83	3.16	<0.01
adult female	−0.04	0.07	23.81	−0.50	1.00
adult male	0.02	0.08	24.79	0.22	1.00
test against null model: *χ*^2^ = 13.36, d.f. = 6, *p* = 0.04 The reference levels are distance initiator 0–1 m and age-sex category juveniles.

## 4. Discussion

We examined the effects of individual, social and contextual factors on the departure process of collective movements in red-fronted lemurs. To this end, we tested the influence of age, sex, proximity at departure and affiliative relationships on departure decisions. Sex and age determined the position in the departure order, with adult females departing more often in the van, juveniles in the centre and adult males in the rear of the group. Joining was a mimetic process guided by both affiliation and distance to the initiator and predecessor.

### 4.1. Strong group cohesion

Red-fronted lemurs followed initiators quickly; in most cases, the whole group joined the movement and initiations rarely failed. Hence, red-fronted lemurs exhibit strong group cohesion. This effective group coordination may be facilitated by the vocal coordination of departure time. Group departures in red-fronted lemurs are preceded by a pre-departure period in which the rate of close calls (grunts) of initiators and followers increases significantly, suggesting that these vocalizations may serve to determine departure time [68]. In addition, the latency from initiation to the last joiner increased with group size, suggesting that a longer duration of the recruitment process might be a cost of living in larger groups.

### 4.2. Age class and sex determine departure order

Age and sex affected the individual decision on departure, which underscores the importance of considering individual variation in the context of collective action [69]. In comparison to adults, juveniles were less likely to be in the van of the departing group and more likely to be in the centre. This observation is in line with the ‘protection theory’ [70,71]: Since the van and rear of a moving group are positions of high predation risk, many species exhibit a ‘protective travel order’ [34] in which juveniles or other vulnerable individuals are situated in the group's centre, as for example, in African buffalo (*Syncerus caffer*) [72], lions (*Panthera leo*) [73] and several primate species (reviewed in [74]).

Adult females were significantly more often in the van of the departing group than males and juveniles. The females' predominance in the van might be due to higher average nutritional needs, since this position may offer better feeding opportunities, as shown in fish schools [40–42] and capuchin monkeys (*Cebus capucinus*) [43]. Adult males left more often in the rear than females, possibly functioning as a rearguard. However, given that there was no difference between adults and juveniles leaving in the rear, the rearguard explanation seems unlikely. Instead, spatial mimetism offers a more likely explanation, as males were generally observed at a greater distance from the initiator than females and would consequently follow later. Thus, the adult individuals’ distribution across departure positions could also be influenced by their spatial distribution. Affiliative mimetism, however, cannot explain the observed assortment by age class and sex in the departure order because affiliative bonds tended to be disassortative, for age classes as well as for sexes.

### 4.3. Affiliative mimetism in joining

Joining was a mimetic process and depended both on affiliative relationships and proximity between individuals. Affiliation had a significant effect on the IDI of dyads, with more strongly affiliated individuals having shorter IDIs and thus joining movements in faster succession. Affiliative mimetism in group departures has also been reported for the closely related brown lemurs (*Eulemur fulvus*) [19]. It is a shared trait between lemurs and anthropoid primates (Tonkean macaques (*Macaca tonkeana*) [17,18], chacma baboons [20], Barbary macaques [22], Tibetan macaques (*Macaca thibetana*) [75]) and diverse other species, including domestic sheep [23,25], domestic geese [27], cattle (*Bos taurus*) [24,25] as well as domestic horses (*Equus caballus*) [26].

### 4.4. Spatial mimetism in joining

The latency to join a movement was determined by spatial mimetism, as initiators and predecessors were followed more quickly by individuals in closer proximity. The influence of proximity was not purely an effect of movement information becoming available later to individuals farther away, since red-fronted lemur groups are highly cohesive and group members are usually in visual or acoustic contact with each other [47] and coordinate departure times vocally [68].

Proximity affecting movement coordination is a mechanism generally associated with large, anonymous groups, where individuals adjust their movements to their closest neighbours, leading to coordinated movements of the whole group [76]. Similarly, spatial mimetism at departure has been shown in anonymous human groups for pedestrian road crossing behaviour, where an individual's likelihood to cross the road increased when their next neighbour started crossing [30]. Still, an effect of proximity on group movements can also be found in individualized groups. In cattle, for example, recruitment success increased when more individuals were in close proximity to the initiator [24]. Likewise, being in the core rather than on the edge of the group had a positive effect on initiation success in white-faced capuchins [77] as well as on recruitment success in rhesus macaques (*Macaca mulatta*), but not in Tonkean macaques [78]. Moreover, spatial proximity can influence an individual's choice of travel direction during group movements. In olive baboons, nearest neighbours predict an individual's location in the short term, whereas over the long term it is determined by affiliative relationships [32].

The influence of inter-individual distance at departure on the timing of departure decisions has rarely been studied (table 3). Where it has been studied, results are in line with our study in red-fronted lemurs. Domestic geese and sheep show affiliative and spatial mimetism, with initiators being followed first by animals in close proximity, which are also their preferred partners [23,29]. Spatial mimetism is a simple and advantageous mechanism because mimicking animals close to oneself allows an individual to benefit from public information while saving on monitoring effort and processing power [79]. Furthermore, it is adaptive, since the neighbours' situation is similar to an individual's own. The information a neighbour is acting upon is thus probably relevant for the individual as well; they could, for example, be aware of a better alternative foraging patch [80]. In addition, if proximity is not circumstantial, but rather the result of preferential association, maintaining this proximity may be the goal itself.

**Table 3.** Overview of studies on collective departures which include a spatial factor. Succession (suc): likelihood to follow an individual (unless latency is specified), leadership: number of successful initiations, order: departure order, recruitment (rec): number of followers. A significant effect is indicated by setting the response in bold. If there is a methodological reason why a factor was not considered, this is marked with * and specified in the comments. For details on the pre-departure and departure behaviours see [68].

species	setting	spatial measure	response spatial measure	label spatial effect	affiliation	response affiliation	sex	age	dominance rank	other	comments	reference
capuchin monkeys (*Cebus capucinus*)	semi-free-ranging	central/peripheral position of initiator	**recruitment**		—		**recruitment** (females)	—	recruitment	initiator departure behaviour (**rec**)		Leca *et al*. [77]
chacma baboons (*Papio ursinus*)	wild	time as nearest neighbours	**succession, order**	effects of dominance rank, spatial association, affiliation and kinship on succession summed up as ‘local rules’	grooming frequency	**succession, order**	leadership	leadership	succession, leadership°, order	kinship (suc, order), pre-departure behaviour (rec)	grooming and time as nearest neighbour correlated; °no general effect of dominance rank, but alpha male most successful initiator	King *et al*. [20]
domestic geese (*Anser domesticus*)	semi-free-ranging	central/peripheral position of initiator neighbours at initiation (<1 m)	**recruitment succession, recruitment**		proximity at rest	**succession**	—*	—*	—	pre-departure behaviour (**rec**), departure behaviour (**rec**)	proximity at rest and neighbours at initiation correlated; *groups consisted of same-sex and -age individuals	Ramseyer *et al*. [29]
domestic sheep (*Ovis aries*)	captive	central/peripheral position of initiator neighbours at initiation (less than 1 m)	**recruitment succession, recruitment**	—	affiliative interactions	**succession**	—*	—*	recruitment	pre-departure behaviour (**rec**)	*groups consisted of same-sex and -age individuals	Ramseyer *et al*. [23]
humans (*Homo sapiens*)	wild	nearest neighbour at initiation	**succession**	—	—*		**succession (following latency)**	—	—*		*anonymous groups by excluding people travelling together	Faria *et al*. [30]
humbug damselfish (*Dascyllus aruanus*)	captive	ranked distance to initiator at initiation	**succession**	local mimetism	—	—	—	—	recruitment	pre-departure behaviour (**rec**)		Ward *et al*. [28]
red-fronted lemurs (*Eulemur rufifrons*)	wild	distance to initiator at initiation distance to predecessor at their departure	**succession (following latency)** **succession (following latency)**	spatial mimetism	time spent grooming and huddling	**succession (inter-departure interval)**	**order**	**order**	—*	interactions between factors	distance at initiation/departure and time spent grooming and huddling correlated; *red-fronted lemurs do not form strict hierarchies	this study
rhesus macaques (*Macaca mulatta*)	semi-free-ranging	central/peripheral position of initiator	**recruitment**		—		—	recruitment	**recruitment**	ID initiator (**rec**), pre-departure behaviour (**rec**), departure behaviour (rec)		Sueur & Petit [78]
tonkean macaques (*Macaca tonkeana*)	semi-free-ranging	central/peripheral position of initiator	recruitment		—		recruitment	recruitment	recruitment	ID initiator (rec), pre-departure behaviour (**rec**), departure behaviour (**rec**)		Sueur & Petit [78]

### 4.5. Interdependence of proximity and affiliation

The effects of affiliation and proximity are generally difficult to discern. In our study, we assessed affiliation independently from the group movement context. Still, dyads that were more closely affiliated were also closer to each other directly before departures. The interdependence of affiliation and proximity is rarely considered in studies on affiliative mimetism. In fact, it is possible that in many cases a measure of affiliation unwittingly acts as a proxy for the distance between individuals and the reported affiliative mimetism could be explained more parsimoniously as spatial mimetism. However, this has no strong implications for the essential role of affiliative relationships in group decisions: either an individual is preferentially following those it has a strong affiliative relationship with, or the individual follows according to proximity, and proximity is the result of preferential association. In the end, affiliative relationships determine the individual's movement decision, whether directly or indirectly [81]. Either way, the cognitive skills necessary for affiliative mimetism, such as individual recognition and differentiated social relationships, would still be required [19]. Despite their interdependence, it is possible to see nuances in the effects of affiliation and proximity on movement decisions. In chacma baboons, group members ‘follow “friends”, but preferentially those friends that are in closest proximity’ [20, p. 1342]. Olive baboons, however, follow neighbours, but preferentially those who are their friends [32].

## 5. Conclusion

In red-fronted lemurs, individual decisions during departures in collective group movements depend on both contextual and social factors, as following is mimetic and influenced by spatial proximity as well as affiliative relationships. However, departure decisions are also influenced by individual traits. This effect is evident in the departure order, which reveals clear positional preferences by different ages and sexes. Working towards uncovering the mechanisms behind the process of group departures, it is thus important to consider individual, social and contextual factors together to understand why an individual chooses to leave at a certain time.

## Supplementary Material

Additional figures and tables

## Supplementary Material

Data & scripts

## Data accessibility

The dataset supporting this article has been uploaded as part of the electronic supplementary material.
