# Supplementary material for: Should I stay or should I go? Individual movement decisions during group departures in red-fronted lemurs
Source: R Soc Open Sci. 2019 Mar 20;6(3):180991. doi: 10.1098/rsos.180991 (PMC6458422; doi:10.1098/rsos.180991)
Supplement: Additional figures and tables [file rsos180991supp1.pdf]

Should I stay or should I go?

Individual movement decisions during group departures in redfronted lemurs

Anna Lucia Sperber, Peter M. Kappeler, Claudia Fichtel

Royal Society Open Science 2019

Supplementary Material

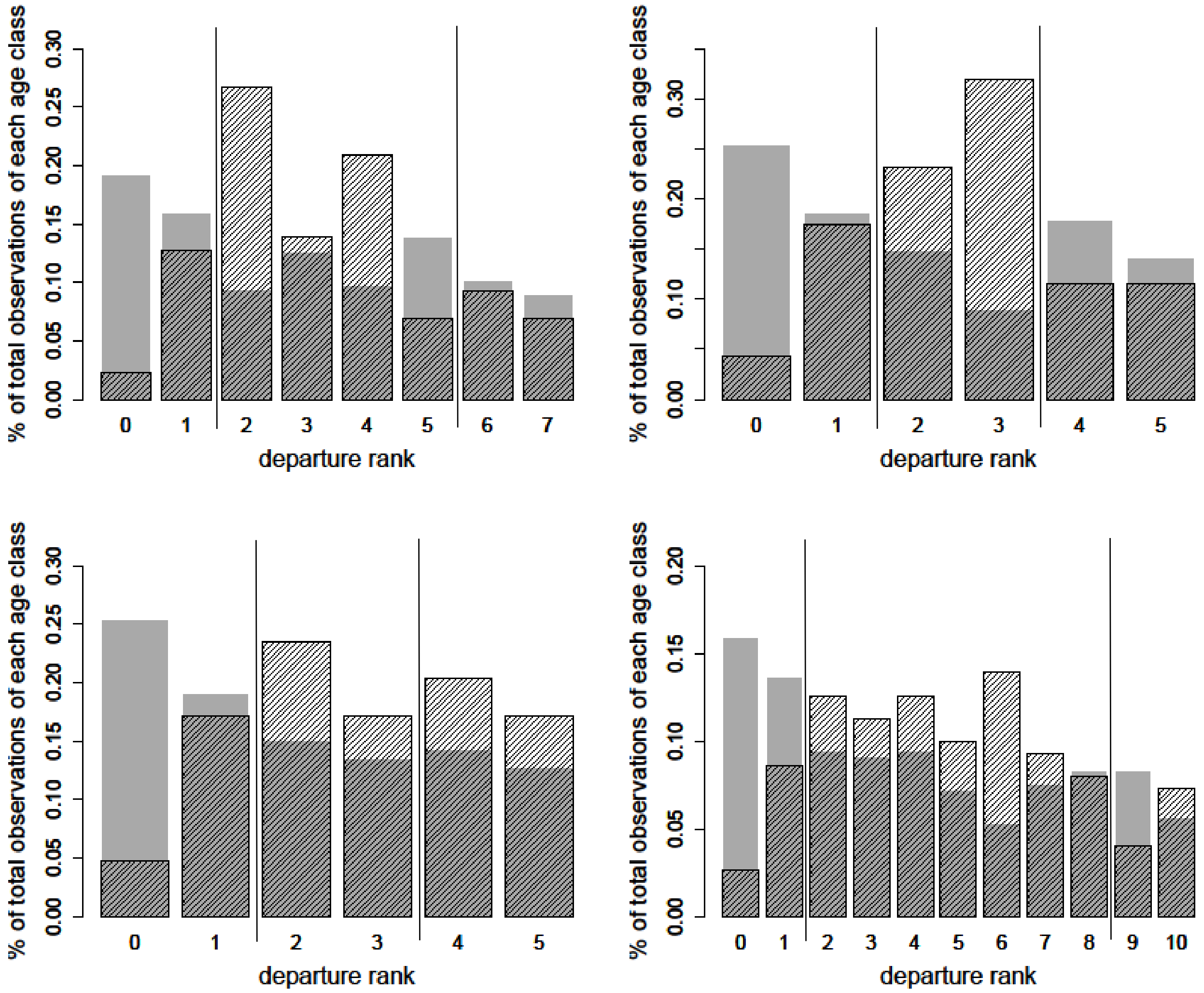

Fig. S1: Distribution of age classes (juveniles and adults) on departure ranks in group A (2 juveniles & 6 adults, top left), B (2 juveniles & 4 adults, top right), F (2 juveniles and 4 adults, bottom left) and J (4 juveniles & 7 adults, bottom right). Proportions were calculated as number of times an age class was observed per rank divided by the total number of observations of the age class. Solid grey bars show adult proportions, hatched black bars juveniles. Rank 0 is the initiator of the movement. Black lines indicate the division into position categories (van, centre & rear) for modelling.

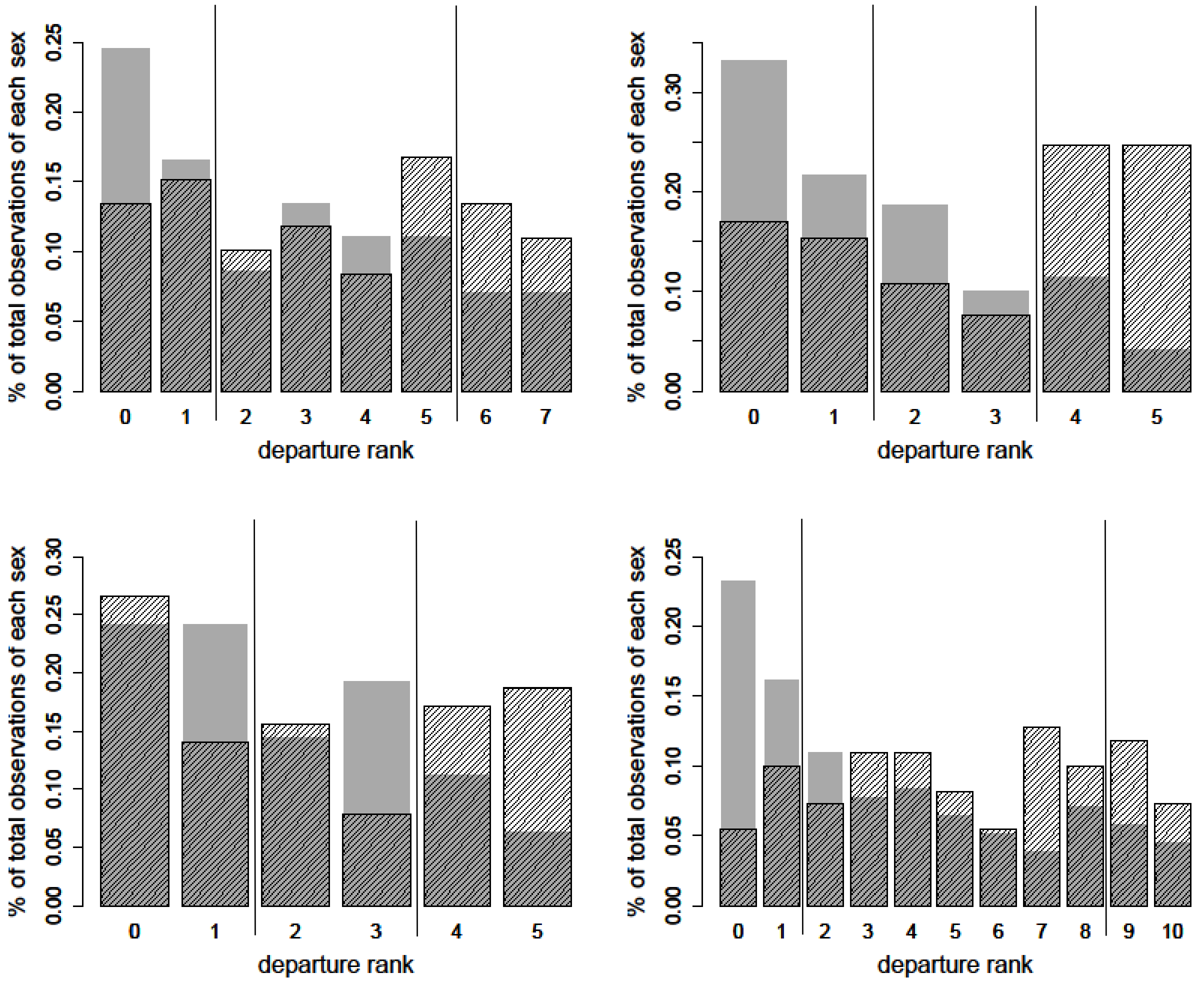

Fig. S2: Distribution of sexes (adult individuals only) on departure ranks in group A (3 females & 3 males, top left), B (2 females & 2 males, top right), F (2 females & 2 males, bottom left) and J (4 females & 3 males, bottom right). Proportions were calculated as number of times each sex was observed per rank divided by the total number of observations of each sex. Solid grey bars show female proportions, hatched black bars males. Rank 0 is the initiator of the movement. Black lines indicate the division into position categories (van, centre & rear) for modelling.

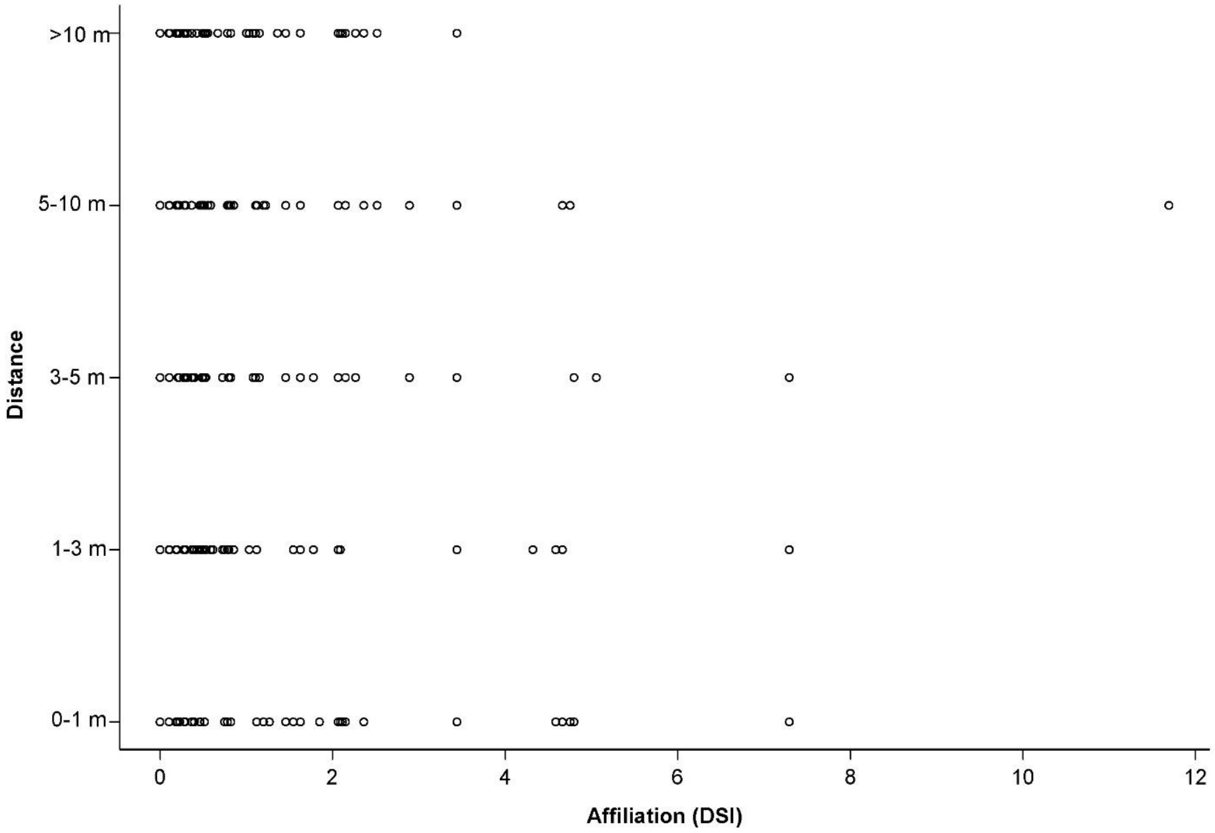

Fig. S3: Proximity at departure depicted as a function of the affiliation index (DSI) of the dyad. Data combined from all four groups (n= 347 observations).

Table S1: Results for the MCMC GLMM regressing a dyad's distances at departure on its dyadic composite sociability index (DSI) and the dyads age-sex combination.

	Post. mean	CI lower	CI upper	Eff.sample size	pMCMC
(Intercept)	4.10	1.86	6.27	48.42	<0.001
DSI	-0.61	-1.10	-0.20	45.50	<0.01
Female-female	0.94	-0.51	2.40	1000	0.68
Female-male	0.90	-0.27	2.25	1000	0.68
Female-juvenile	-0.01	-1.21	1.15	1000	1.00
Male-male	1.17	-0.42	2.68	1000	0.67
Male-juvenile	-0.07	-1.31	1.05	1000	1.00
Group B	0.58	-0.89	2.32.	1000	1.00
Group F	-0.29	-1.78	1.39	826.51	1.00
Group J	-0.02	-1.67	1.52	764.50	1.00

Cutpoints:

	Post. mean	CI lower	CI upper	Eff.sample size
Cutpoint distance 1	3.80	2.13	5.65	22.89
Cutpoint distance 2	6.45	3.70	9.69	19.28
Cutpoint distance 3	10.19	6.13	15.41	18.14

The reference levels are age-sex combination juvenile-juvenile and group A.

Table S2: Effect of age class and sex on departure position. Results from multiple comparisons of the effects from the LMM.

	Estimate	CI lower	CI upper	Std. Error	t value	Pr(>|t|)
female van-male van	0.12	0.00	0.24	0.04	2.81	0.05
female centre-male centre	0.00	-0.12	0.13	0.04	0.10	100.00
female rear-male rear	-0.13	-0.25	-0.01	0.04	-2.99	0.03
female van-juvenile van	0.21	0.09	0.33	0.04	4.93	< 0.0001
female centre- juvenile centre	-0.15	-0.27	-0.03	0.04	-3.37	0.01
female rear- juvenile rear	-0.05	-0.17	0.07	0.04	-1.19	0.84
male van- juvenile van	0.09	-0.03	0.22	0.04	2.06	0.28
male centre- juvenile centre	-0.15	-0.27	-0.03	0.04	-3.39	0.01
male rear- juvenile rear	0.08	-0.05	0.20	0.04	1.76	0.46

Test against null model: F= 11.09, df= 14, p<0.001

Table S3: Assortativity by age and sex (juvenile, adult male, adult female. Mixing matrices and weighted assortativity coefficient (AC) by group.

**Group A**	**female**	**male**	**juvenile**	**$a_i = b_i$**
**female**	0.09	0.11	0.22	0.42
**male**	0.11	0.05	0.09	0.25
**juvenile**	0.22	0.09	0.02	0.33

AC ± SE: -0.28 ± 0.08

**Group B**	**female**	**male**	**juvenile**	**$a_i = b_i$**
female	0.02	0.17	0.12	0.31
male	0.17	0.13	0.11	0.41
juvenile	0.12	0.11	0.05	0.28

AC ± SE: -0.21 ± 0.13

**Group F**	**female**	**male**	**juvenile**	**$a_i = b_i$**
**female**	0.02	0.11	0.28	0.40
**male**	0.11	0.00	0.09	0.20
**juvenile**	0.28	0.09	0.02	0.39

AC ± SE: -0.5 ± 0.14

**Group J**	**female**	**male**	**juvenile**	**$a_i = b_i$**
**female**	0.05	0.17	0.14	0.36
**male**	0.17	0.06	0.07	0.31
**juvenile**	0.14	0.07	0.12	0.33

AC ± SE: -0.16 ± 0.08

Table S4: Effect of age class and sex on distance to the initiator. Results from multiple comparisons of the effects from the LMM.

	Estimate	CI lower	CI upper	SE	t value	Pr(>|t|)
female <1m - male <1m	0.02	-0.14	0.19	0.06	0.40	0.99
juvenile <1m - female <1m	0.04	-0.13	0.20	0.06	0.62	0.96
juvenile <1m - male <1m	0.06	-0.10	0.23	0.06	0.99	0.83
female >10m - male >10m	-0.17	-0.34	-0.01	0.06	-2.74	0.04
juvenile >10m - female >10m	0.06	-0.11	0.22	0.06	0.93	0.86
juvenile >10m - male >10m	-0.11	-0.28	0.05	0.06	-1.77	0.33

Test against null model: F= 2.12, df= 17, p= 0.01

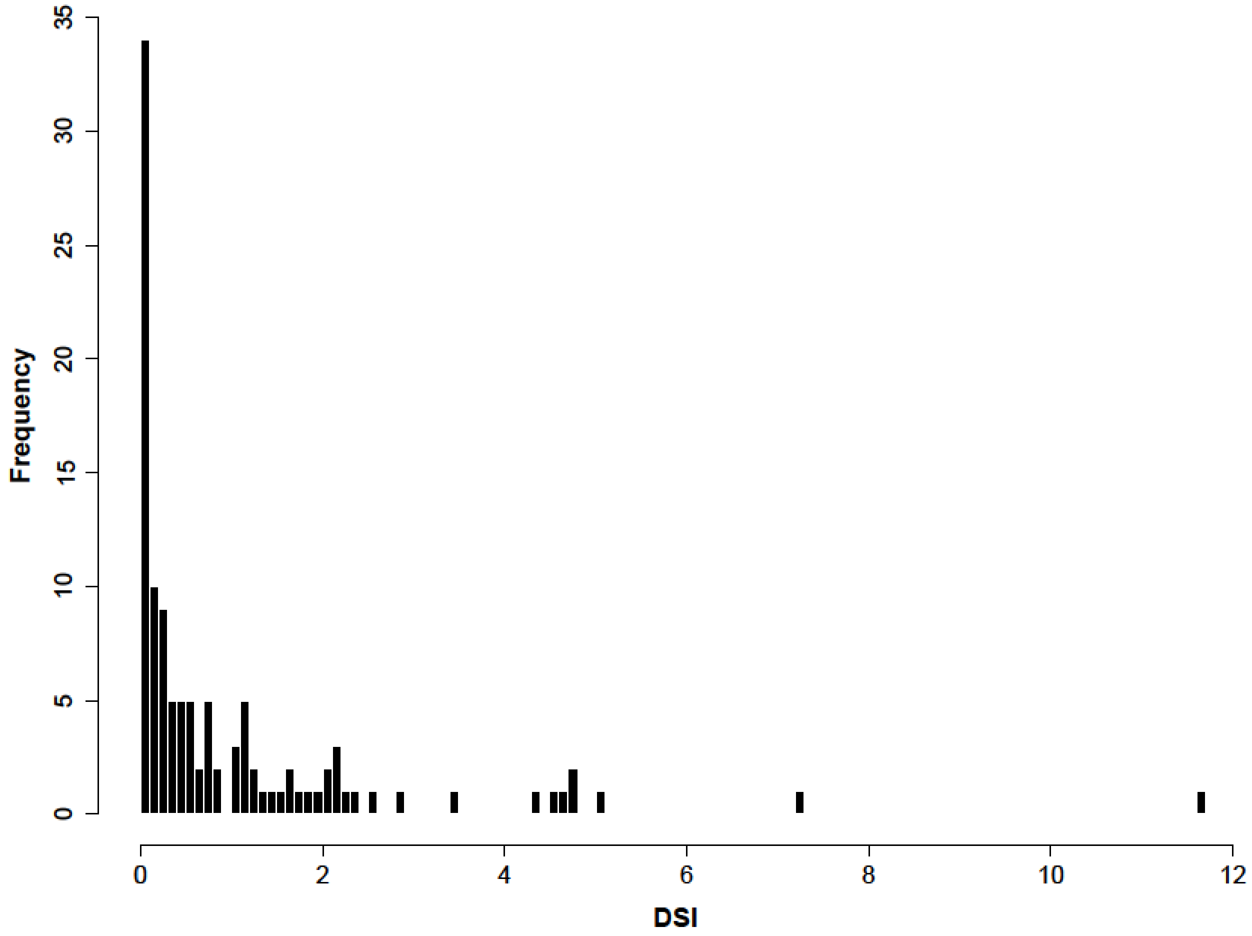

Fig. S4: Distribution of dyadic sociability index values. Data pooled across groups (n = 113 dyads), but DSI calculated within groups. (mean = 1, median = 0.38, range = 0 - 11.69).
